# Engaging Scientific Diasporas in STEAM Education: The Case of Science Clubs Colombia

**DOI:** 10.3389/frma.2022.898167

**Published:** 2022-06-28

**Authors:** Bryann E. Avendano-Uribe, Andres Lombana-Bermudez, Laura V. Flórez, Elisa Chaparro, Adriana Carolina Hernandez-Morales, Jorge Archbold, Juan Camilo Buitrago-Casas, Ana Maria Porras

**Affiliations:** ^1^ScienteLab, Bogota, Colombia; ^2^HIT Lab NZ, University of Canterbury, Christchurch, New Zealand; ^3^Department of Civil and Natural Resources Engineering, University of Canterbury, Christchurch, New Zealand; ^4^Berkman Klein Center for Internet and Society, Harvard University, Cambridge, MA, United States; ^5^Department of Communication, Pontificia Universidad Javeriana, Bogotá, Colombia; ^6^Department of Plant and Environmental Sciences, University of Copenhagen, Copenhagen, Denmark; ^7^The Evergreen State College, Olympia, WA, United States; ^8^Department of Civil and Environmental Engineering, Universidad del Norte, Barranquilla, Colombia; ^9^Space Sciences Laboratory and Physics Department, University of California, Berkeley, Berkeley, CA, United States; ^10^Pruitt Family Department of Biomedical Engineering, University of Florida, Gainesville, FL, United States

**Keywords:** educational policy, Colombian scientists, scientific diasporas, STEAM education, scientific vocations, K-12 education

## Abstract

Currently, there is limited insight on the role that scientific diasporas can play in STEAM education in Latin America. Here, we present the Science Clubs Colombia (Clubes de Ciencia Colombia-SCC) program, a pioneering STEAM capacity-building initiative led by volunteer scientists to engage youth and children from underserved communities in science. The program brings together researchers based in Colombia and abroad to lead intensive project-based learning workshops for young students in urban and rural areas. These projects focus on channeling the students' technical and cognitive scientific aptitudes to tackle challenges of both local and global relevance. The program provides high-quality STEAM education adapted to communities' needs and articulates long-lasting international collaborations using the mobility of the Colombian diaspora. The program's success is tangible via its sustained growth and adaptability. Since its first version in 2015, 722 volunteer scientists living abroad or in Colombia have collaborated to create 364 clubs with the participation of 9,295 students. We describe elements of the SCC program that lead to a scalable and reproducible outcome to engage science diasporas in STEAM education. Additionally, we discuss the involvement of multiple stakeholders and the generation of international networks as potential science diplomacy outcomes. The SCC program strengthens the involvement of Latin American youth in science, demonstrates the potential of engaging scientific diasporas in science education, and enriches connections between the Global South and the Global North.

## Introduction

Preparing youth for current and foreseeable global challenges should be a top priority, and an endeavor reaching all geographical and social contexts. In particular, curricula in STEAM (Science, Technology, Engineering, Arts and Mathematics) education are at the core of this task. Through high-quality STEAM education, students acquire crucial skills such as critical thinking, perseverance, creativity, innovation, and collaboration. These essential competencies increase access to job stability, higher income, and productivity, which are vital to a nation's growth (Vuorikari et al., 2016) and to address pressing challenges worldwide. Furthermore, a highly educated workforce is paramount to drive economic development and build a knowledge-based economy (NSF, 2014; UNESCO, 2017). Thus, it is crucial for nations to implement education policies that develop critical skills for problem-solving and build research capacity for their countries (Greenbaum and Hajjar, 2017).

*Science Clubs Colombia* (Clubes de Ciencia Colombia, 2022,^1^, henceforth SCC) is a grassroots educational program created and developed by volunteer scientists to address this challenge. Inspired by the pioneering work by *Science Clubs Mexico*, the program was born in 2015 led by Colombian scientists as a hands-on learning experiential initiative for youth. Nowadays, the program's mission is to mobilize the scientific diaspora for STEAM education in Latin America, contributing to high-quality STEAM education in underserved communities (Franco et al., 2019). The model has grown to include Colombian and non-Colombian volunteers located worldwide as part of the Latin American initiative *Science Clubs International*^2^.

**Figure 1:**
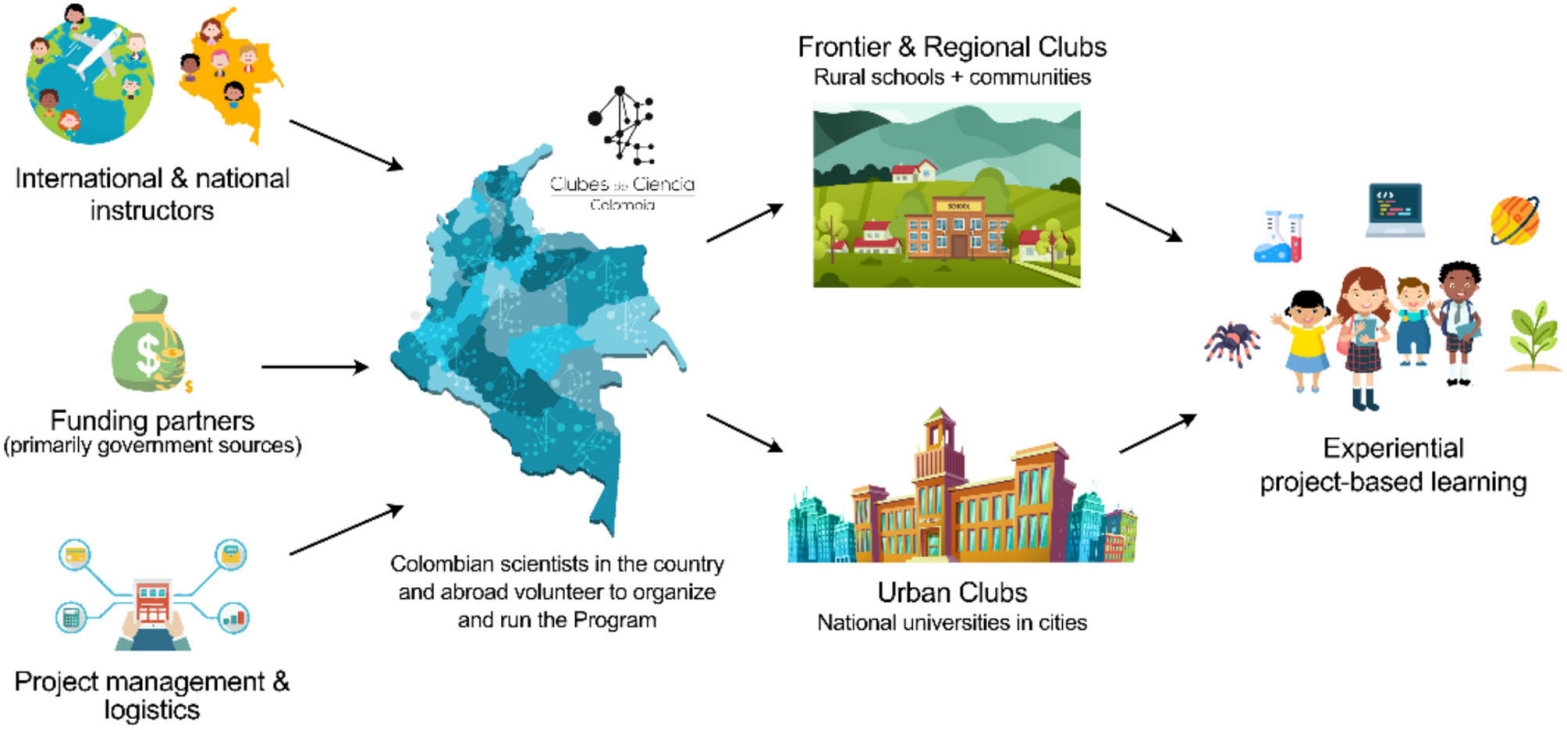
Structure of the Science Clubs Colombia (SCC) program. The SCC program is a synergy between Colombian scientist living in the country and abroad, international and national instructors, funding agencies, a logistic team, and several other stakeholders that come together to deliver the different science clubs in both rural and urban areas of the country.

In Colombia, the program is sustained by a network of volunteers who work with support from both private and government entities (Figure 1). Currently, SCC is affiliated with ScienteLab, a non-profit organization that seeks to strengthen science and technology appropriation processes in different contexts, focusing on youth and children in Latin America. Also, SCC has established important relationships with governmental institutions that provide financial support, yet not on a permanent basis. As a program, the goals of SCC are (1) to increase access to high-quality STEAM education for Colombian children and youth by engaging the scientific community in the country and abroad, and (2) to facilitate connections between the diaspora and local scientists and communities.

These goals are addressed by the program through the execution of “Science Clubs”, science workshops delivered to youth and children across the Colombian territory. As described in detail in Section Science Clubs—The SCC Approach, these science clubs are led by teams of scientists based in Colombia and abroad. To make these Science Clubs possible, the volunteer team, consisting of Colombian scientists based both in the country and abroad, is essential. They donate their time to articulate several tasks, including fundraising, recruitment and selection of instructors and students, media and communications management, finance administration, material purchase, logistics, and workshop design support. The volunteers work alongside logistic teams to ensure the science clubs take place as planned. This task force constitutes a synergistic cooperation, connecting and engaging members of the Colombian scientific diaspora. Importantly, as part of the more extensive network of *Science Clubs International*, these alliances transcend a single country and have become increasingly more robust between the different countries that take part in the project in and beyond Latin America.

In this report, we first outline the motivation and context in which the SCC model has been developed. We then describe the foundational elements of the program and the primary outcomes in the past seven years. Furthermore, we expand on the potential implications of engaging scientific diasporas as a potential science diplomacy strategy to advance STEAM education in Latin America.

### The Potential of Science Diasporas Within STEAM Education

Higher education is a strong driver of emigration in developing countries, especially among STEAM professionals (Dodani and LaPorte, 2005). Since Latin America overall invests less in STEAM, the access to funding and career options is lower for researchers in the region (Valenzuela-Toro and Viglino, 2021). The migration of scientists and STEAM higher education students to high-income nations is thus a significant challenge affecting human capital building in Latin America. This phenomenon, generally known as brain drain, results in the loss of some highly trained or qualified individuals. The social impact of displacement has not only reduced the region's economic potential (Cerovic and Beaton, 2017), but it has also tapered technological innovation in the region (Lozano-Ascencio and Gandini, 2012). This situation is reflected in the region's low output of patents and publications (Ciocca and Delgado, 2017) and diminished access to high-quality education (Busso and Messina, 2020).

As a country with a long-standing internal conflict and deep social disparities, Colombia has historically had a high emigration rate, including many people who are earning or have earned their graduate degree in a STEAM field (Docquier and Marfouk, 2004; Özden, 2006; Docquier, 2014). Among these, on average only one of every three scientists trained abroad returns to the country, as most of them report not encountering the conditions to continue with their research or find opportunities for professional development (Meyer et al., 1997; Didou Aupetit and Gérard, 2009). As many scientists remain abroad, there is a loss of potential expertise and role models in STEAM careers for the Colombian youth. Thus, STEAM education could benefit and be highly nourished by the participation of science diasporas. Recent reports on science diplomacy for Latin America propose strengthening the scientific system by reconnecting scientists from the region living abroad (Gual-Soler, 2021) and promoting the internationalization of the scientific community and efforts throughout collaboration between nations (Lopez-Verges et al., 2021b).

### Context: Education Inequality in Colombia

Colombia is a country of divides and one of the most unequal countries in the world (Alvaredo et al., 2018; Busso and Messina, 2020; World Bank, 2021). Economic gaps in income, territorial disparities in access to basic infrastructure and social disparities in access to opportunity are among the multiple inequalities affecting the life chances and wellbeing of Colombian children and youth. Inevitably, learning and educational opportunities and outcomes are heavily affected under these conditions. In the long run, educational inequality also deepens the divides between social groups and territories. Recent indicators of student academic attainment and achievement, as well as schools' access to resources and teacher performance, reveal that Colombian children and youth from rural areas and low socioeconomic backgrounds have limited educational opportunities and poor academic outcomes (OECD, 2016a, 2019; Fundación Empresarios por la Educación FexE, 2021; World Bank, 2021).

In the last decades, Colombia has developed several policies to improve its educational system and expand its coverage. Despite these efforts, economic disparities, spatial divides, and other markers of disadvantage reinforce the still persistent educational inequalities (OECD, 2016b; World Bank, 2021). Additionally, the increase in public investment in education and the expansion of coverage have not been accompanied by systematic improvements in the quality of education. Several analyses of student performance in PISA tests and SABER national tests demonstrate that inequalities in students' academic performance are associated with their social ties. These ties include students' socioeconomic status, the type of school they attend (private or public), and the school's geographic zone (urban or rural) (Duarte et al., 2012; Gamboa and Londoño, 2015; Ramos Lobo et al., 2016; OECD, 2019, 2016a; Gomez-Gonzalez et al., 2021). Key factors causing educational disparities include the low quality of teacher training, the lack of a national curriculum, disparities in school funding—public and rural schools generally receive less funding—and selection bias in teacher-to-school assignments (Duarte et al., 2012; Fundación Empresarios por la Educación FexE, 2021; Gomez-Gonzalez et al., 2021; World Bank, 2021).

### Rural Education and the Challenges of a Violent History

The urban-rural spatial divide needs special consideration because it reinforces extreme educational inequalities in Colombia. The territorial gap has a long history of marginalization of rural areas and their populations, particularly minority ethnic groups (Afro-Colombians and indigenous people), excluding them from access to basic services, employment, quality education, and other economic, social and learning opportunities (UNDP, 2011). Despite the efforts made by the government to close the urban-rural gap, the divide continues to evolve in complex ways. In 2020, the share of rural Colombians living in multidimensional poverty was 37.1% compared to 12.5% of urban dwellers (DANE, 2021). Likewise, educational inequality is more significant in rural areas. Indicators of educational attainment, for instance, reveal largely unfair conditions. Compared to students in urban centers, populations in rural areas receive fewer years of schooling (MEN, 2015) and have lower school enrollment and higher illiteracy rates (DANE, 2018). The added and devastating effects of climate disasters and the COVID-19 pandemic exacerbate this disparate scenario in many communities (Busso and Messina, 2020).

Furthermore, Colombian rural territories have been the most affected by the armed conflict (Gómez Soler, 2017), the longest civil war on the continent. The consequences range from political instability and altered economic and agricultural activities to direct violence, human displacement, and recruitment of children and teenagers into armed forces. As an example of the extreme and direct effects, between 1990 and 2020, there have been 331 violent seizures or attacks in educational institutions by armed forces in Colombia (Bernal et al., 2021). Inevitably, these factors profoundly impact the possibilities and perspectives of Colombian rural youth.

### Perspectives for Moving Forward

There is an inherent difficulty in transforming the multifactorial inequity outlined above, underlining the pressing need to enhance and diversify education opportunities that promote social mobility. Notably, occupational perspectives for low socioeconomic levels and rural communities are usually strongly constrained. There are not only lower expectations for higher education (Radinger et al., 2018), but there is also a lack of exposure to diverse options that allow children and youth to explore their interests and potential. In particular, science education is lagging behind. In Colombia and Latin America, there is much room for improvement in enhancing STEAM experiences for early ages, and this is particularly pronounced when considering equal opportunities for different genders, ethnicities, and socioeconomic groups. The SCC program considers that (1) accessing high-quality STEAM education is fundamental to address many of the inequities highlighted before, and (2) engaging the highly-qualified Colombian STEAM workforce in both the country and the diaspora, as well as international allies, is crucial to achieve equity in education.

## Science Clubs—The SCC Approach

A Science Club is an intensive, project-oriented, 1-week workshop focused on developing technical and cognitive abilities in a wide array of research areas in STEAM fields [Clubes de Ciencia Colombia, 2022 (see text footnote 1); Science Clubs International | Science Education, 2022 (see text footnote 2); Franco et al., 2019]. The Science Clubs aim to serve as a platform for young students to experience the world of research: conception of ideas, design, execution of experiments, and communication of results. Each Science Club is led by an international instructor, who is a graduate student or professional researcher affiliated with an international institution, in collaboration with an equally qualified local instructor based in Colombia (Figure 1). Project-based learning (PBL) is the essence of the instructional approach of the Science Clubs. This approach supports extended inquiry processes, engaging students in real-world problem solving, and providing meaningful and active learning opportunities (Jones et al., 2004; Helle et al., 2006; Capraro et al., 2013). In the Science Clubs, students are actively involved in the learning process through exploration, experimentation, and collaboration. Combining STEAM and PBL, Clubs are designed to foster creativity, critical thinking, and the development of skills students can apply to solve problems in their communities and local contexts. This approach has been applied across the *Science Clubs International* network in multiple countries, including Colombia (Bravo-Mosquera et al., 2019), Mexico (Lengeling and Jinich, 2019), Bolivia (Ferreira et al., 2019), Brazil, Peru, Ecuador, Paraguay, and Spain (Hernandez-Lopez et al., 2021).

The Science Clubs carried out in Colombia are geared toward teenagers ranging from 13 to 17 years old, who are students of public middle schools and high schools. Given the country's vast cultural and social diversity, SCC divides the Clubs in three large categories—Urban, Regional, and Frontier (Figure 1). Most Urban Science Clubs have historically been co-organized with the “Tecnoacademias” Program from the Colombian National Technical Training Service (SENA). They take place in cities and occur in partnership with local universities that lend their facilities to host the workshops. In contrast, the Regional and Frontier versions take place in rural regions across the country. The Frontier Science Clubs focus on working with students in remote and borderland communities located in departments like Amazonas, Nariño, Vichada, and La Guajira. These Clubs have traditionally been financed through an alliance with the Colombian Ministry of Science and Technology. Finally, the Regional Clubs' primary goal is to serve rural communities all over the national territory in partnership with local authorities and organizations. The Regional and Frontier Clubs look for meeting needs and interests specific to each location and community (Buitrago-Casas et al., 2020). SCC achieves this objective by promoting the exploration of local talent and resources, acknowledging existing ancestral and communal knowledge, and encouraging the adaptation and appropriation of new knowledge while sparking curiosity and creativity in students.

## Results and Program Outcomes

### Reach of Science Clubs Colombia

**Figure 2:**
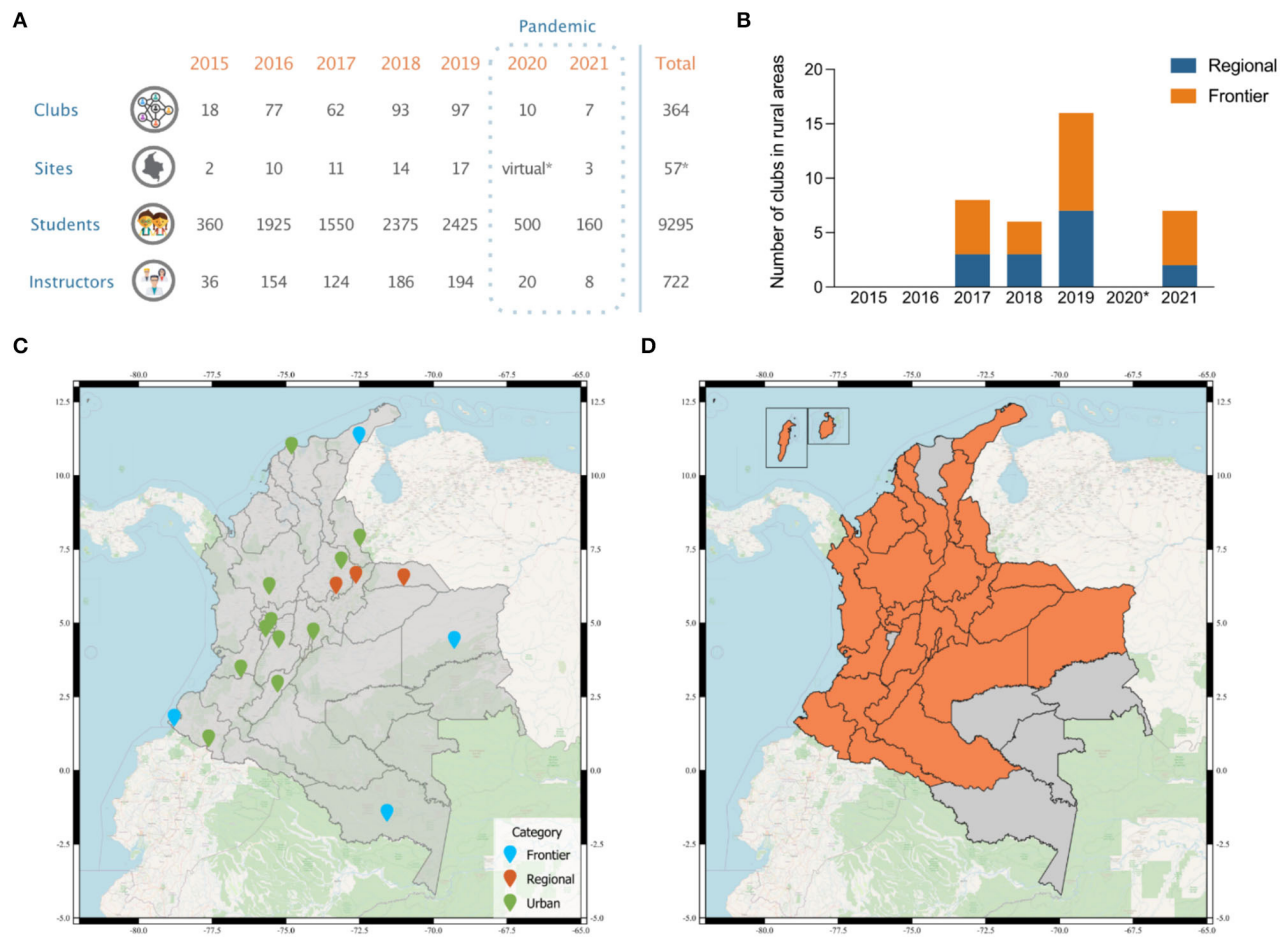
Impact of the program (2015–2021). **(A)** Number of Science Clubs, participants, and sites during the seven years of the program operation in Colombia. *Denotes the virtual edition of the program that ran in 2020 due to the COVID-19 pandemic. The 2021 edition of the Program consisted of a hybrid implementation of the Clubs—“Science Calls You” edition. **(B)** The number of Science Clubs that have taken place in rural communities through the Regional and Frontier versions of the program. **(C)** Map of the geographical distribution of Science Clubs sites in Colombia throughout the history of the program. Colors indicate urban or rural (including both regional and frontier) locations. **(D)** Colombian departments reached during the 2020 Clubs at Home edition of the Program.

Since its first edition in 2015, SCC has organized 364 Clubs impacting the lives of over 9,000 students across 57 sites in Colombia (Figures 2A,B). Whether in urban or rural areas, most of the students come from public schools, and many from low-socioeconomic status households, with limited access to high-quality education. Led by 722 instructors, the Clubs have covered a wide variety of STEAM topics: food science, biomedical engineering, artificial intelligence, conservation, agriculture, applied math, astronomy, biotechnology, social sciences, genetics, nuclear physics, and many others. SCC has reached students from 28 out of 32 Colombian departments (Figures 2C,D), despite the many inequities students face in both urban and rural settings. In particular, 37 Science Clubs have occurred in rural communities during the Regional and Frontier versions of the Program (Figures 2B,C). These communities are located in areas at risk of failing infrastructure, food insecurity (e.g., Guajira), and different levels of involvement in armed conflict (e.g., Amazonas, Arauca, Nariño, and Vichada).

### Engaging the Colombian Diaspora

**Figure 3:**
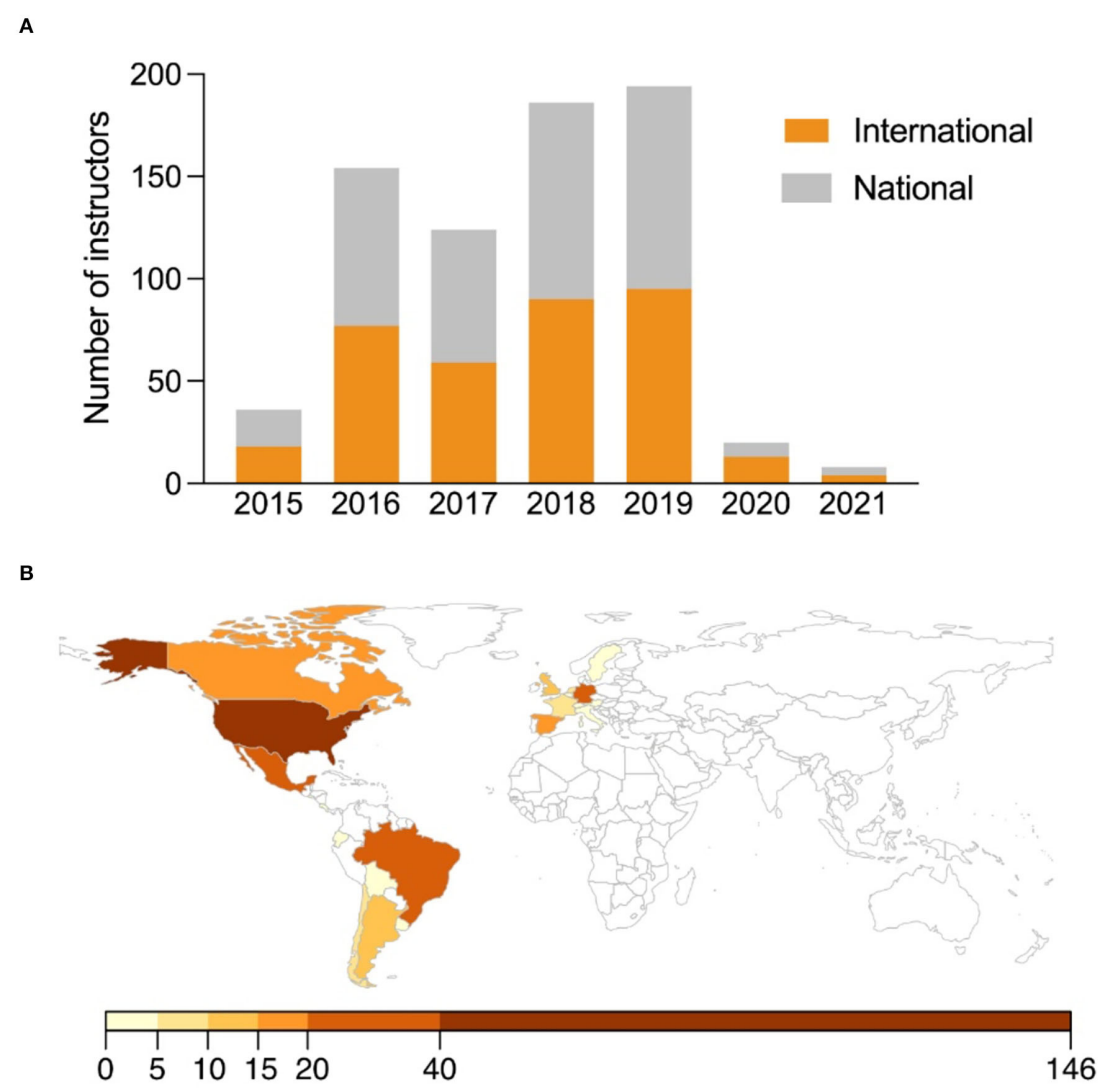
International instructor involvement and distribution. **(A)** Total number of national and international instructors involved in the Program annually from 2015 to 2021. **(B)** Global distribution of international instructors defined by location of work or study during the year their corresponding Club took place.

SCC targets the science diaspora and its networks to connect local scientists and students with international researchers. To achieve this goal, at least one instructor based at an international institution is involved in each club. The volunteers coordinating the program advertise the call through social media and international channels reaching researchers abroad. Applications are received via an online platform, and a systematic process is conducted to select the scientists who will travel to Colombia as instructors. To date, 356 international and 366 national instructors have taught a Science Club with consistent international and national participation throughout every edition of the Program (Figure 3A). In total, 49.3% of instructors worked or studied abroad the year they participated in the Program. These instructors were based in 22 different countries across North, Central, and South America; and Europe (Figure 3B; Supplementary Table 1). Notably, 75% of international instructors worked or studied in 5 countries: Spain (5%), Mexico (6%), Germany (9%), Brazil (11%), and the United States (44%). This diverse geographic representation allows the program to incorporate a wide variety of perspectives and teaching styles.

**Figure 4:**
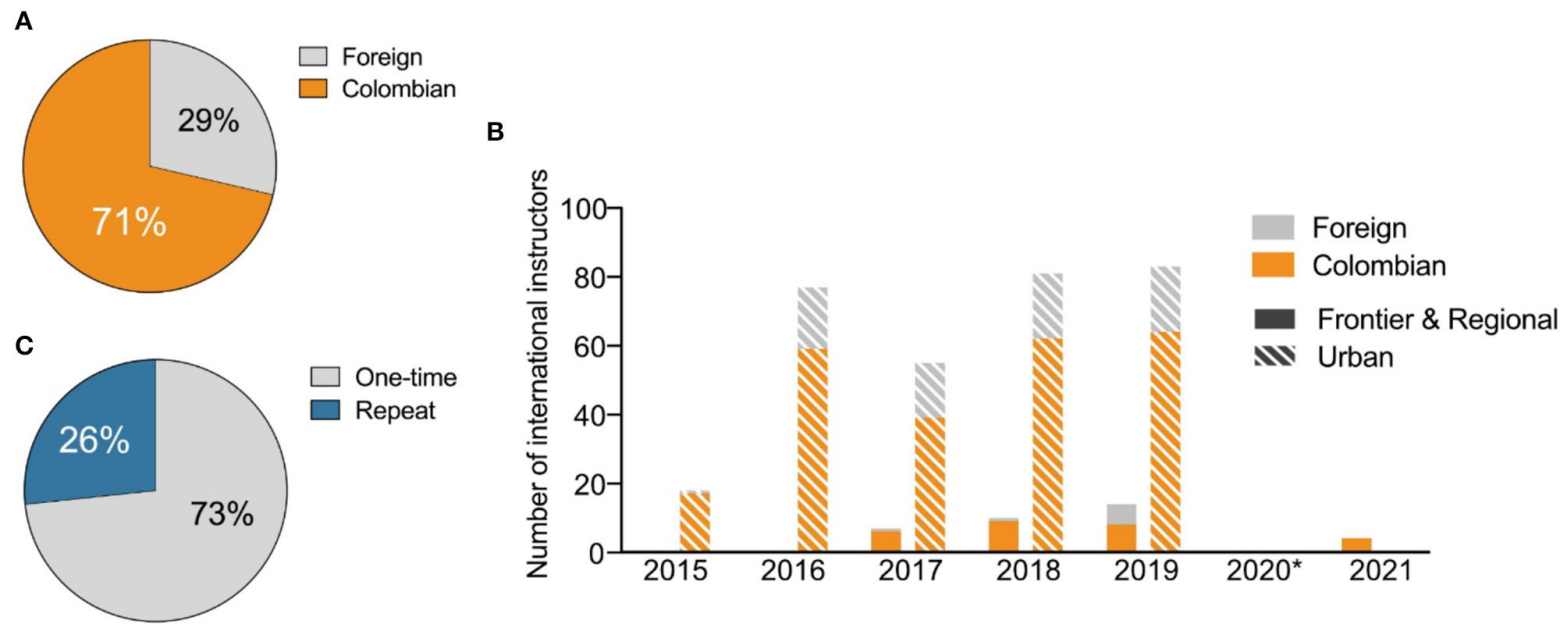
Engagement of the Colombian diaspora. **(A)** Percentage of international instructors that are Colombian citizens vs. citizens of other countries (“foreign”). **(B)** Visualization of the proportion of international instructors involved in Frontier, Regional, and Urban versions of SCC who are Colombian citizens vs. foreign. **(C)** Percentage of international instructors who have participated in the Program for multiple years.

While the international instructors were located worldwide, 71% of them were part of the Colombian diaspora (Figure 4A). That is, they are Colombian citizens who now work and study outside of Colombia. This distribution highlights the high interest amongst the diaspora to return and contribute to the development of the country. An essential aspect of the program is the opportunity for instructors to travel to the locations where the Clubs take place (Figure 2C). Thus, scientists from the Colombian diaspora can witness the critical issues affecting local contexts and learn from their interactions with students and communities. Visiting the cities or regions where the Science Clubs are held, the instructors have the opportunity to get to know a new community or place, and to immerse themselves in the culture and reality of the locals. These opportunities create spaces that foster the exchange of knowledge, experiences, and worldviews, making it clear that science is a universal language and an effective tool to generate alliances and strengthen the social fabric. The instructors travel with the intention of teaching yet find themselves possibly also being students and raising their awareness about realities that are starkly different from their day-to-day lives. These experiences are vital for Regional and Frontier Clubs, which historically attract international instructors who are primarily Colombian citizens (Figure 4B; Supplementary Figure 1). At the same time, each Science Club experience is also a networking opportunity between instructors, school teachers, local leaders, and students. As a result, many instructors of the diaspora leave the Program with a renewed desire to engage in solutions to Colombian's most pressing issues. In fact, 27% of international instructors have returned to teach a Science Club one or more times (Figure 4C).

### Adapting to a Changing Global Landscape

SCC relies on international collaboration to achieve its goal of providing access to high-quality STEAM education. This project-based learning program was designed to be conducted in person. During the first 5 years, the program grew in numbers and impact without interruptions. As a result, the program was heavily affected by the onset of the COVID-19 pandemic. Health and safety concerns, the redistribution of government funding to face the pandemic, international travel restrictions, and local mobility restrictions led to an interruption of traditional programs. Nonetheless, the program was able to adapt and create a new “Clubs at Home” version of the Science Clubs. In this version, instructors designed 4 week-long modules, including pre-recorded lectures and worksheets with experiments to be performed at home with readily available materials. Due to the lack of geographical constraints, these Clubs were open to students all over the country. We were therefore able to reach a higher volume of students, 500 to be precise, with only 10 Clubs (Figures 2A,B). Additionally, we introduced the methodology to students from 12 new departments, primarily in urban areas (Figure 2D). However, this first foray into remote learning also highlighted the disadvantages and lack of infrastructure faced by students in rural areas of the country.

The lack of equipment, wireless connectivity, and in some cases, reliable electricity severely limited the ability of former participants in the Regional and Frontier Clubs to access any virtual platform. To address these challenges, the program pivoted once again in 2021 with an innovative hybrid solution for these populations—the “Science Calls You” Clubs (Avendaño-Uribe, 2021). A few instructors from the “Clubs at Home” version were asked to come back and reuse previously created videos and content. Students received personalized kits consisting of a modified old-school cellular phone (not smartphones), learning guides, worksheets, notebooks, and all materials necessary to carry out all experiments and activities. Students and instructors would communicate through calls using a voice-only platform and the phones throughout the 4 weeks of the Clubs. As usual, 50% of the instructors were international (Figure 3A). The selection of the clubs' topics matched the needs of each community in the fields of agriculture, social and data science, math, and hydrology. The targeted communities were located in the Isipha and Wayma communities in La Guajira and Carcasí, Santander, for a total of 7 Clubs and 160 students (Figures 2A,B). Some of the instructors were able to travel to La Guajira in person.

The “Science Calls You” version sought to implement a more sustainable approach for these communities through four key strategies to continue the program in future editions. (1) Obtaining funding to perform upgrades to the local infrastructure where necessary that would outlast the Clubs. (2) Local teachers helped conduct the Science Clubs by providing space in their classrooms and guiding the students toward the successful execution of all proposed activities. (3) Study guides and worksheets were designed to fit the cultural norms and practices of the region and created with important contributions in ethno-education by local teachers. (4) Some of the Clubs' activities were aimed at providing long-term solutions for these communities. For example, students and teachers established a garden in their indigenous school at the Wayuu community of Ishipa, located in La Guajira, a department plagued by droughts and food insecurity. These efforts once again underscore the importance of and potential for collaboration between communities in Colombia and the scientific diaspora.

## Discussion

### Possibilities for the Scientific Diaspora Through Science Clubs Colombia

*SCC* has been successful in mobilizing the science education ecosystem in the country. Thanks to more than 300 organizations and over 700 scientists working together, it has been possible to promote the participation of scientists from different nations in high-quality science education in the Latin American region and Colombia. These articulated efforts have demonstrated that the science diaspora can be actively connected to STEAM education in Colombia.

In this report, we describe how SCC promotes high-quality STEAM education adapted to the needs and context of the country while contributing to robust international scientific networks and collaborations as a potential diplomatic outcome. We illustrate the expansion of the program deliberately oriented toward reducing unequal opportunities by reaching diverse communities and a broad geographic range within the country. Additionally, we discuss several characteristics of the program and its development that underline its potential value for strengthening international bonds. Those international relationships are cornerstones of potential diplomatic structures and reveal the role of the scientific diaspora in STEAM education.

First, we show the consistent participation of researchers based abroad that have teamed up with researchers living in Colombia throughout the history of the program. This collaboration suggests a significant contribution to strengthening international ties between scientists. Second, the participation records indicate that most international instructors are Colombians, revealing interconnectedness and commitment among the Colombian scientific diaspora. Third, the sustained growth in the number of instructors and the probability that they repeat the experience (26%) indicates that the program is attractive and engaging for Colombian and non-Colombian international researchers. Fourth, the involvement of different global regions, including Europe, North and South America, shows the broad-reaching impact of the Science Clubs project. This program and similar initiatives propose a valuable link between STEAM education and science diplomacy that should tackle social, political and technological issues in Latin America and worldwide.

### Harnessing Science Diplomacy to Integrate STEAM Education in Latin America

Science diplomacy has included a wide range of topics and negotiation themes with the goal of tackling current challenges such as food security, climate change, Antarctic governance, and public health (Turekian et al., 2014). In several nations, science diaspora communities already play an integral part in facilitating solutions to such global challenges (Burns, 2013). Within Latin America, experts on science diplomacy have recommended the “articulation of networks of scientists abroad to strengthen national science systems and foster brain circulation” (Gual-Soler, 2021). Nonetheless, the inclusion of the science diaspora into STEAM education as a potential outcome for science diplomacy has received minimal attention in the region. The SCC Program is based on the premise that organized scientific diasporas could benefit national STEAM education and could promote scientific collaboration between nations.

Although SCC is not a science diplomacy initiative, the program is built on interactions between stakeholders from different nations—multilateral organizations, research institutes, universities in Colombia and abroad, organizations in the public and private sector, civil society, and other institutions. It engages the Colombian scientific diaspora, local scientists and institutions, and international allies to provide high-quality science education to historically marginalized communities. These activities require new network building with the purpose of providing STEAM education. Furthermore, they may lead to a secondary diplomatic outcome—strengthening international relations to impact science education on a national and regional scale. Additionally, members of the scientific diaspora who participate in the program stay connected throughout spin-off projects, and formalize partnerships and alliances with both local and national governments that may foster long-term collaboration between international stakeholders. These activities lead to new network building within STEAM education, research, and development.

Following the creation of the International Mission of Wise Men and Women (Pavas and Arzola de la Peña, 2019) and the establishment of the first Ministry of Science, Technology, and Innovation (Minciencias), Colombia has set the new strategic objective of positioning, making visible, and articulating the sectors of science, technology, and innovation at the international level. The plan is to create a national science diplomacy strategy with nodes in nine Latin American countries (including the neighbors Brazil, Panama, and Peru) (Gual-Soler, 2021). This governmental strategy focuses on training and capacity building in science diplomacy, both within the government and other entities/actors. Notably, it demands greater coordination between the diaspora and the Colombian scientific communities. The positioning of Colombia in South-South alliances is also envisaged, considering its entry into the OECD in April 2020 and its potential to support countries in the region with lower capacities (Gual-Soler, 2021). In the long-term, the SCC Program is positioned to become a key partner in this mission given its existing engagement with the Colombian diaspora, governmental institutions, and international partners.

We argue that articulating science education into the diplomacy agenda can start with mobilizing the science diaspora from the Global North and South to support STEAM education in less-favored areas. There is an additional need to increase efforts tackling the multiple inequities that have intensified in recent years in Latin America, particularly in Colombia. As the analysis of SSC reveals, this program has enabled the mobilization of the science diaspora, promoting scientific vocations, and promoting science education as a pillar of transformation in society. The SCC model has been replicated in different contexts of Latin America, Spain, and the USA; it can inspire similar efforts in other regions where science education is still a shortage for underrepresented communities such as Africa, Asia, and Pacific-Oceania.

Particularly, the implementation of the Frontier and Regional version of our program highlight the need for STEAM education in rural areas and the potential for engaging scientific diasporas in the process. Little research has been done on the impact of mobilizing human capital, including established researchers and graduate students, as promoters of scientific vocations or STEAM education in rural areas. Importantly, through this scientific experience the students gain insight on how STEAM training could foster transformation in their communities.

### Key Challenges

A connected and active science diaspora engaged in STEAM education can lead to scientific diplomacy outcomes. Keeping science diaspora engaged into STEAM education promises long-term benefits for high-quality education, such as the Science Clubs Colombia program. However, this promising approach bears some challenges that deserve special attention, as outlined below.

First, human capital is a major challenge for the execution and sustainability of the program. The project's rapid expansion gives rise to intrinsic demands for large-scale project management. Major time and efforts are invested in recruitment, logistics, communication, fundraising, and pedagogical support. Given that the program's management is taken over by a group of <15 volunteers who are full-time scientists or doctoral candidates, consistent and extended commitment of team-members is extremely challenging. Incentives in the form of logistic and/or financial backup for the researchers promoting grassroots initiatives like *Science Clubs* would guarantee the long-term sustainability of these programs.

A second key challenge is sustaining a robust and reliable financing scheme. Historically, a combination of public and private institutions has funded the program. However, none of these entities provide constant financial support, meaning that fundraising is a yearly task for the volunteer team. Incoming contributions rely on rigorous proposal writing and the availability of funding bodies each year. Considering the large scale of the program, this also results in tight budgets and the need to cut down on features of the program, like paid educational staff or longer duration of the projects, which could further boost pedagogic quality. In response to this challenge, in 2021, *Science Clubs Colombia* aimed at incorporating local teachers in the workshops and designed follow-up activities that provide continuity to the learning experience. However, financial sustainability remains a challenge.

Another opportunity for improvement is attracting researchers from a broader geographic range to participate as international instructors. This challenge has intrinsic difficulties associated with language—the workshops are held in Spanish—, and is naturally biased toward countries that have a historical connection to the program or where a large number of Colombians seek higher education, as is the U.S.A. However, Science Clubs and the associated scientific network would benefit from further strengthening participant diversity. Notably, Latin America has created academic exchange and mobility impacting the scientific ecosystem (Lopez-Verges et al., 2021a). An increasing number of Colombians engage in doctoral and post-doctoral research experiences abroad, including countries such as Spain, Mexico, Argentina, Germany, Chile, France, and Peru (Peña Castañeda, 2019), which should be reflected in the program. The feasibility to support researchers from various countries, primarily those more geographically distant, relies on fund availability and therefore goes hand-in-hand with the challenges described above.

## Concluding Remarks

This report describes elements of a grassroots science education program that leads to a proven, scalable and reproducible outcome of connected science diasporas and their engagement in STEAM education. The model of SCC program can be replicated in different contexts and children will benefit from a high-quality scientific education program that leads to international collaboration and long-term networking for promotion of STEAM careers.

This is the first brief research report where more than 700 scientists over 7 years have participated in constructing a network that impacts children and young students in rural areas of Colombia. This paper intends to inspire other STEAM programs that convene the science diaspora to work hand in hand in collaborations to improve the quality of education in Latin America. It is also a call for future science diplomacy frameworks in the region to include STEAM education initiatives that mobilize the science diasporas as a critical global strategy to increase scientific and technological capacity. Additionally, we exemplified the importance of engaging science diasporas in science education as a mobility experience that potentially could benefit Global South and Global North countries. Finally, engaging science diasporas to temporarily return to their countries of origin to support rural education in disadvantaged communities has potentially a long-term impact on the scientific vocations of children and youth—an area of interest outside of the scope of this paper that we will investigate further. Thus, promoting learning and collaboration between local and international networks impacts the Latin American scientific ecosystem.

The promise of a future where the science diaspora actively contributes to STEAM education, mobilizing, and collaborating with different national educational programs is not far from reality if science diplomacy programs and public policy recommendations include STEAM education in the agenda. Integrating science diasporas in STEAM education could potentially have outcomes that impact the long-term dynamics of science diplomacy and could help to bridge educational inequalities. The idea is to include highly trained international scientists in the national education system so that schools become small-scale science laboratories, where teaching, learning, and research potentially improve experiential science education. We seek an education where teachers, community and students can articulate efforts to tackle cutting-edge problems using STEAM skills and abilities to permeate public education systems.

## Data Availability Statement

The data analyzed in this study is subject to the following licenses/restrictions: the dataset of volunteer scientists is not openly available and we reserve the right to share it. In our research we do not use names or personal identifications. We have taken the adequate provisions to protect the privacy of subjects and to maintain confidentiality of data. Requests to access these datasets should be directed to ScienteLab/Clubes De Ciencia Colombia, conocimiento@scientelab.org.

## Author Contributions

BA-U conceived the idea, led the submission and writing process, and he coordinated the team to structure the paper. AP created most tables and figures, wrote the approach and results section, and led the rewriting of the subsequent drafts. AL-B wrote the context and included scientific references for the academic content of the paper. LF led the edition, part of the structure, and wrote many contributions along the paper, including introduction, context, results, and discussion. EC conducted data mining and organization of the raw data from 7 years of the program. JA created the maps and contributed to finalized versions. AH-M contributed to the introduction of the paper. JB-C helped with final formatting, edition, and proofreading. All authors contributed to the article and approved the submitted version.

## Funding

During the past 7 years, *Science Clubs Colombia* had the financial support of several Colombian and foreign institutions. Here we mention all those who have provided us with economic resources for the execution of this project: Ministry of Science, Technology and Innovation of Colombia, (MinCiencias- Before Colciencias), Administrative Department of Science, Technology and Innovation- Ondas Program, Colombian National Technical Training Service (SENA), COLPATRIA Bank, Colombian Association for the Advancement of Science (AVANCIENCIA), Parque Explora, Harvard Faculty of Arts and Sciences - Department of Molecular and Cellular Biology, Gobernación de Antioquia, Gobernación de Atlántico, Ruta N, Colpatria Group, Sapiencia - Alcaldía de Medellín, Alcaldía de Oiba, Manuelita Foundation, Motorola Foundation, IBM Corporate Citizenship Program, La Cooperativa de Ahorro y Crédito de Profesores (Cooprofesores), Lyda Hill Philanthropies through the IF/THEN Program, RVG-IPS, Six continents travel agency, Corpoemprende, Postobón, and Sur Sur Innova (Chile). Our Clubs would not have been possible without your support.

## Conflict of Interest

BA-U, AL-B, LF, EC, AH-M, JA, and JB-C were volunteers with ScienteLab. The remaining author declares that the research was conducted in the absence of any commercial or financial relationships that could be construed as a potential conflict of interest.

## Publisher's Note

All claims expressed in this article are solely those of the authors and do not necessarily represent those of their affiliated organizations, or those of the publisher, the editors and the reviewers. Any product that may be evaluated in this article, or claim that may be made by its manufacturer, is not guaranteed or endorsed by the publisher.

## Footnotes

^1^ https://www.clubesdeciencia.co

^2^ https://www.scienceclubsint.org

## Supplementary Material

Supplementary Figure 1 Percentage of international instructors in Frontier/Regional and Urban versions of the Science Clubs Colombia Program who are Colombian citizens.

Supplementary Table 1 Distribution of international instructors per country.
