# Supplementary figures and images for: Engaging Scientific Diasporas in STEAM Education: The Case of Science Clubs Colombia
Source: Front Res Metr Anal. 2022 Jun 28;7:898167. doi: 10.3389/frma.2022.898167 (PMC9274008; doi:10.3389/frma.2022.898167)

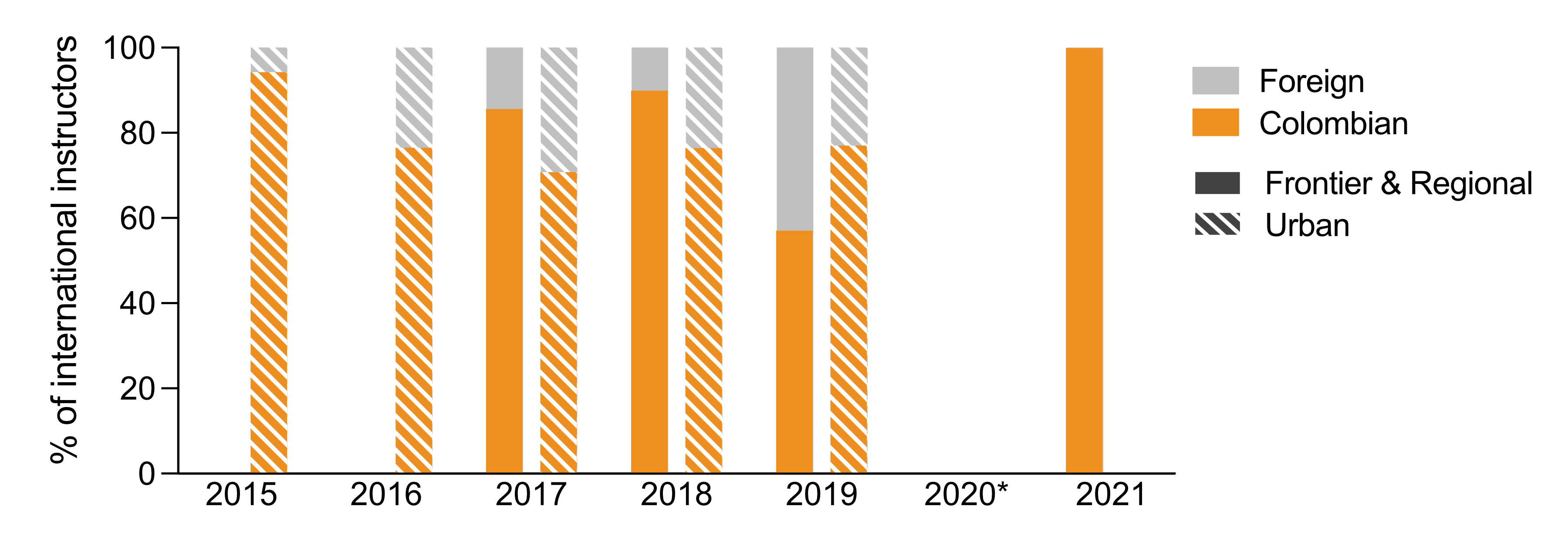

Supplement: Supplementary Figure 1 — Percentage of international instructors in Frontier/Regional and Urban versions of the Science Clubs Colombia Program who are Colombian citizens. [file Image_2.JPEG]

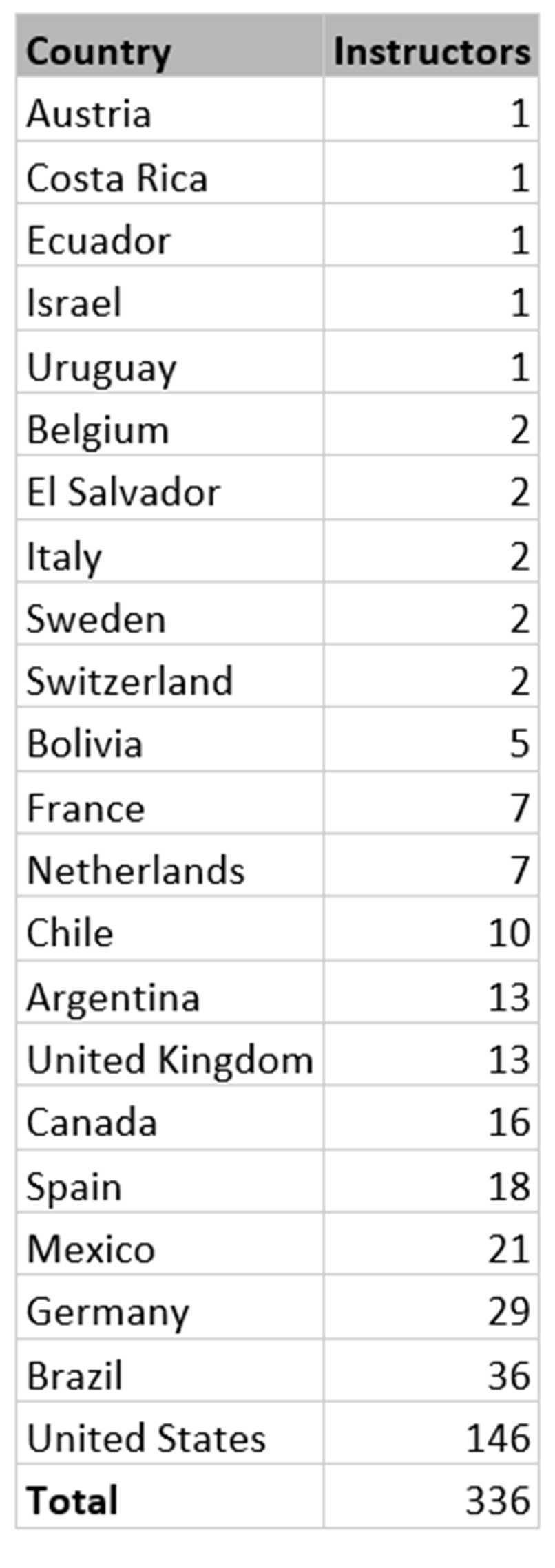

Supplement: Supplementary Table 1 — Distribution of international instructors per country. [file Image_1.PNG]
